# The Exosome-Mediated Epigenome: Non-Coding RNA and mRNA-Coding Networks in Microbiome–Cellular Communication, Inflammation, and Tumorigenesis Along the Oral–Gut–Lung Axis

**DOI:** 10.3390/epigenomes9040052

**Published:** 2025-12-16

**Authors:** Beatriz Andrea Otálora-Otálora, César Payán-Gómez, Juan Javier López-Rivera, Luisa Fernanda Patiño-Unibio, Sally Lorena Arboleda-Mojica, Claudia Aristizábal-Guzmán, Mario Arturo Isaza-Ruget, Carlos Arturo Álvarez-Moreno

**Affiliations:** 1 Grupo de Investigación INPAC, Unidad de Investigación, Fundación Universitaria Sanitas, Bogotá 110131, Colombia; claristizabal@unisanitas.edu.co; 2 Dirección Académica, Universidad Nacional de Colombia, Sede de La Paz, La Paz 111016, Colombia; cepayang@unal.edu.co; 3 Grupo de Investigación INPAC, Specialized Laboratory, Clinica Universitaria Colombia, Clínica Colsanitas S.A., Bogotá 111321, Colombia; jjlopez@colsanitas.com; 4 Grupo de Investigación INPAC, Department of Internal Medicine, Clinica Universitaria Colombia, Clínica Colsanitas S.A., Bogotá 111321, Colombia; minternacuc@colsanitas.com; 5 Grupo de Investigación INPAC, Facultad de Medicina, Fundación Universitaria Sanitas, Bogotá 110131, Colombia; sally.arboleda@unisanitas.edu.co; 6 Keralty, Sanitas International Organization, Grupo de Investigación INPAC, Fundación Universitaria Sanitas, Bogotá 110131, Colombia; misaza@keralty.com; 7 Infectious Diseases Department, Clinica Universitaria Colombia, Clínica Colsanitas S.A., Bogotá 111321, Colombia; calvarez@colsanitas.com

**Keywords:** exosomes, epigenome, transcriptome, transcription factors, long-noncoding RNAs, cancer- and inflammatory-related cells membrane receptors, microbiota

## Abstract

**Background/Objectives**: The oral–gut–lung axis represents a dynamic system where exosomes carrying mRNAs and non-coding RNAs might help to regulate microbiota and human cell crosstalk to establish transcriptional regulatory networks controlling cellular biological processes and signaling pathways. **Methods**: We conducted a comprehensive transcriptomic analysis to characterize the molecular cargo of extracellular exosomes in the context of gut and lung cancer. **Results**: By analyzing gut and lung exosomes cargo with our previous transcriptomic studies from tumoral and inflammatory tissues, we found that exosomes can transport key RNAs that codify specific receptors that facilitate pathogenic interaction with microorganisms and RNAs that are part of interacting gene and transcriptional regulatory networks that control the function of differentially expresses genes, all involved in biological processes like cell cycle, plasticity and growth regulation, invasion, metastasis, microenvironmental remodeling, epigenetic, and microbial and immunological modulation, during the unlocking of phenotypic plasticity for the acquisition of the hallmarks of cancer in the oral–gut–lung axis. **Conclusions**: Exosomal RNA regulation of transcriptional networks represents a pivotal axis in the interplay between inflammation and cancer, offering opportunities for innovative diagnostic and therapeutic approaches.

## 1. Introduction

The oral–gut–lung axis represents a dynamic system where exosomes carrying coding RNAs (mRNAs) and non-coding RNAs (ncRNAs) might help to regulate microbiota and human cell crosstalk to establish transcriptional regulatory networks by mediating cellular biological processes and signaling pathways. Extracellular vesicles (EVs) are lipid bilayer-enclosed membranous structures secreted by cells in humans, other mammals, and plants, as well as microbial organisms, classified as apoptotic bodies (50 nm to 5 μm), microvesicles, ectosomes or microparticles (50 nm to 1 μm), and exosomes (30 to 150 nm) [1]. Exosomes carry different nucleic acids and proteins necessary for cell-to-cell communication via interaction on the cell surface, to initiate intracellular signaling pathways and/or transfer cargo molecules related to both physiological and pathological processes, such as cancer growth, migration, progression, invasion [2], and angiogenesis [3], as well as tumor immune microenvironment remodeling [4]. Cancer cells exchange exosomes with stromal cells to create a protumor microenvironment, increasing tumor invasion and proliferation [5]. Fibroblast-derived exosomes reprogram cancer cell metabolism, providing de novo “off-the-shelf” metabolites through exosomal cargo, which increases glucose uptake, lactate secretion, and reduces oxygen consumption rate [6]. Tumor-derived exosomes can reshape distant microenvironments, as pre-metastatic niches, driving organ-specific metastasis, through the induction of endothelial cell branching and inflammation in the perivascular niche [7]. Likewise, Gram-positive bacteria can make membrane vesicles that include microbial proteins, nucleic acids, and peptidoglycan, detected by innate immune receptors known as pattern recognition receptors in host epithelial cells, and induce autophagy, related tumor development, progression, metastasis, and immunological responses [8]. Gram-negative bacteria produce outer membrane vesicles that bud from the outer membrane, where they play essential roles in diverse bacterial life events, including regulation of microbial interactions, pathogenesis promotion, stress responses, and biofilm formation [9]. Fungal EVs contain proteins, carbohydrates, pigments, nucleic acids, toxins, and other bioactive molecules in the intercellular matrices or on the surface, which are transported to the extracellular environment and at long distances to exert immunogenic effects [10]. Viruses share many characteristics with EVs, like the use of cellular machinery, the packaging of substrates, and secretion for replication, assembly, and egress. Moreover, both RNA and DNA viruses, enveloped or not, use the extracellular vesicles pathway to secrete their viral particles, proteins, and nucleic acids, as well as host elements that benefit viral infection, while extracellular vesicles regulate viral infections by transporting immunomodulatory molecules and viral antigens to initiate antiviral immune responses [11]. Virus EVs modulate many aspects of the immune system, leading to both antiviral and pro-viral responses that can drive a variety of autoimmune and chronic inflammatory diseases and cancers [12]. EVs released by endothelial cells, smooth muscle cells, and immune cells have been implicated in vascular dysfunction, inflammation, and remodeling transport bioactive molecules, including microRNAs, proteins, and lipids, which can impact recipient cells in the pulmonary vasculature, influencing vasoconstriction, smooth muscle cell proliferation, and endothelial dysfunction [13].

Exosomal mRNAs encoding cytokines (e.g., IL-1β, TNF-α) or enzymes (e.g., COX2) can be delivered to recipient cells, enhancing the inflammatory response, and can be translated into recipient immune cells, promoting the production of inflammatory mediators and sustaining chronic inflammation, which is a cancer-promoting factor [14]. Exosomal mRNAs encoding oncogenic proteins, such as MYC or VEGF, can transform recipient cells or promote angiogenesis. Those delivered to stromal or endothelial cells support tumor growth and proliferation by activating mitogenic pathways [15]. miRNAs carried by exosomes target mRNAs in immune cells, regulating key inflammatory signaling pathways such as NF-κB, MAPK, and JAK/STAT, which suppress anti-inflammatory signals and skew responses toward a tumor-supportive microenvironment [16]. Tumor-derived exosomes often carry miRNAs that suppress tumor suppressor genes like HOXD10, promoting metastasis [17]. The exosomal miR-200 family is an important regulator of epithelial–mesenchymal transition (EMT), enabling metastasis [18]. Exosomal circRNAs can attract miRNAs, indirectly influencing inflammatory gene expression by regulating miRNA availability, and regulating tumor cell metastasis through the activation of signaling pathways and affecting the tumor microenvironment [19]. CircRNAs like circ_0004277 delivered via exosomes affect gene expression by interacting with transcription factors (TFs) or RNA-binding proteins in recipient cells, mediating DNA damage response and maintaining genomic stability to act as a tumor suppressor gene [20]. Exosomal lncRNAs are related to epigenetic reprogramming, influencing chromatin states in immune cells, altering transcriptional profiles to sustain inflammation, modulating cytokine transcription, promoting persistent inflammatory responses conducive to tumor progression and metastasis [21]. Exosomal lncRNAs can also enhance the expression of genes associated with cancer stem cell-like properties [22]. The RNA cargo of exosomes creates a feedback loop between inflammation and tumorigenesis, promoting the persistent activation of inflammatory pathways, creating a tumor-supportive microenvironment [23]. Therefore, exosomal RNAs can become diagnostic and prognostic biomarkers for cancer and inflammatory diseases, while the modulation of the mRNA-coding and non-coding RNA content of exosomes, and the blocking of their uptake by cells, can become a treatment in inflammation-induced tumorigenesis scenarios [24].

In our previous transcriptomic analyses of gastric, colon, and lung inflammatory and tumoral diseases [25,26], transcriptional regulatory networks (TRNs) were studied under the regulation of the microbiome network in the oral–gut–lung axis. The gene annotation analysis of upregulated genes highlighted the cell receptors that might be involved in host–microbiome crosstalk and the control of specific deregulated signaling pathways in inflammatory and cancer-related cells. Now, we want to extent our transcriptomic analysis considering the key function of tumor-derived exosomes for the communication between inflammatory and cancer-related cells with the microbiota in the oral–gut–lung axis that might be related to the activation of signaling pathways and biological processes involved in the unlocking of phenotypic plasticity for the acquisition of the hallmarks of cancer during the establishment of inflammatory and tumorigenic processes in the oral–gut–lung axis.

## 2. Results

### 2.1. Transcriptomic Analysis of Overexpressed RNAs Related to Extracellular Exosomes from Circulating Whole Blood Plasma or Serum in Tumoral Diseases of the Gut–Lung Axis

In LC patients, serum exosomes have 2520 overregulated RNAs (Table S1). According to the DAVID annotation analysis, only 507 RNAs are known to be linked with extracellular exosomes. LC blood exosome (LCEXO) RNAs are generally related to the function of the cells’ membrane, cytosol, endoplasmic reticulum, ribosomes, extracellular region, lysosomes, membrane raft, endosomes, Golgi apparatus, mitochondrial outer membrane, and nuclear envelope. There are several LCEXO RNAs, including transcriptional regulators (CEBPB, IRF1, JUN, and MYC) and membrane receptors (TLRs, MHCI, MHCII, TNFs, IFNs, ICAMs, FAS, integrins, and Fc gammas) involved in signaling pathways related to host and microbiota crosstalk like coronavirus disease COVID-19, *Epstein–Barr virus* (EBV), *Mycobacterium tuberculosis*, *staphylococcus aureus*, *Leishmania*, *influenza A virus*, *shigella*, *Escherichia coli*, *bordetella pertussis*, *salmonella*, *measles virus*, *toxoplasma gondii*, *human cytomegalovirus* (HCMV), *yersinia*, *human T-cell leukemia virus 1* (HTLV-I), and *kaposi sarcoma-associated herpesvirus* (KSHV) infections. LCEXO RNAs are also involved in other signaling pathways like ribosome, phagosome, complement and coagulation cascades, antigen processing and presentation, inflammatory, innate and adaptive immune response, HIF-1, TNF, cell adhesion molecules, extrinsic and intrinsic apoptosis, Fc gamma R-mediated phagocytosis, cell surface receptor, cytokine-mediated, and pathways in cancer. LCEXO RNAs are also related to the regulation of cytoplasmic translation, focal adhesion, and epigenomic reprogramming processes like hydroxylation, acetylation, and phosphorylation, viral processing, transcription, gene expression, life cycle, and entry into host cell, cell migration, communication, proliferation, development, differentiation, and homeostasis, angiogenesis, phosphate metabolic process, exocytosis, and endocytosis.

In GC patients, blood exosomes have 1999 overregulated RNAs (Table S2). According to DAVID, no RNA is known to be linked with extracellular exosomes. Several GC blood exosome (GCEXO) RNAs encode for glycoproteins, which are generally related to the function of the cells’ mitochondrion, mitochondrial large ribosomal subunit, cell membrane, and endoplasmic reticulum membrane. GCEXO RNAs are related to G-protein-coupled receptors, biosynthesis of cofactors, WNT, and metabolic signaling pathways. GCEXO RNAs are also related to transmembrane signaling receptor activity with ESR1, GPRASP2, and NR1I2. GCEXO RNAs are related to transducer and signal transduction activity, most of them G-protein coupled (GCPRs) membrane receptors and transmembrane signal receptors, as well as four TFs (ESR1, HLX, NR1I2, and STAT3). GCEXO RNAs are also related to defense response to Gram-negative bacterium, the establishment or maintenance of cell polarity, intestinal immune network for IgA production, antifungal humoral response, positive regulation of cell migration, stem cell division, as well as epigenetic reprogramming with the positive regulation of protein phosphorylation, and protein K48-linked ubiquitination.

In CC patients, plasma exosomes have 615 overregulated RNAs (Table S3). According to DAVID, only 40 RNAs are known to be linked with extracellular exosomes. Several CC plasma exosomes (CCEXO) RNAs are glycoproteins, generally related to the function of the cell surface, extracellular matrix, microtubules, cytoskeleton, and endocytic vesicle membrane. CCEXO RNAs are related to signaling pathways like HSV1, Toxoplasma, and hepatitis C (HCV) infection; WNT; pathways in cancer; basal cell carcinoma; mRNA surveillance; the pluripotency of stem cells; G-protein coupled receptor protein; cytokine-mediated; regulated secretory; and cell surface receptor. CCEXO RNAs are also related to DNA replication, recombination, and repair, transcription regulation, RNA splicing, cytokine activity, vesicle docking involved in exocytosis, cell fate differentiation, communication, and junction assembly, mesenchymal–epithelial cell signaling, nuclear receptor coactivator activity, negative regulation of epithelial cell apoptotic process, and GTPase activity. CCEXO RNAs are also related to the regulation of the metabolic process, viral life cycle, and viral process, as well as epigenetic reprogramming through the negative regulation of histone modification, protein complex disassembly, protein acylation, depolymerization, and phosphorylation.

**Figure 1:**
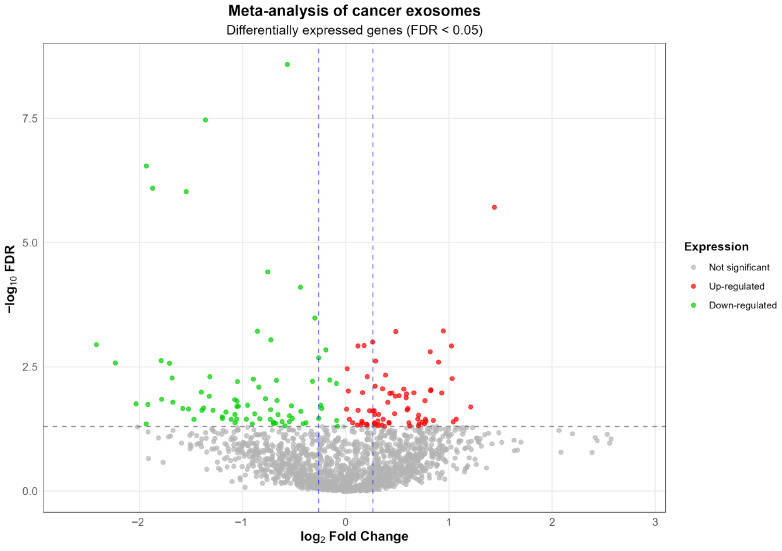
Volcano plot of the meta-analysis of exosome-derived RNA-seq data from plasma of patients with lung, gastric, and colon cancers. Each point represents a gene. Red points indicate significantly up-regulated genes (FDR < 0.05 and log_2_FC > 0), green points indicate significantly down-regulated genes (FDR < 0.05 and log_2_FC < 0), and gray points indicate non-significant genes. The horizontal dashed line represents the statistical significance threshold (FDR = 0.05). The vertical dashed lines indicate log_2_ fold change thresholds of ±0.261, corresponding to a 1.2-fold change in linear scale.

The whole transcriptome meta-analysis of blood exosomes for lung, gastric, and colon cancer identified a shared and unique molecular signature among these three tumor types (Figure 1). The analysis highlighted 73 overregulated RNAs, of which 23 are lncRNAs, 4 are pseudogenes, and 7 are membrane receptors, along with another 33 mRNAs. Most of the gene coding proteins contain a stretch of amino acids present in multiple copies, or have a signal sequence, a peptide usually present at the N-terminus of proteins and which is destined to be either secreted or part of membrane components, or are modified by the formation of a bond between the thiol groups of two peptidyl-cysteine residues, or contain one or more covalently linked carbohydrates of various types, i.e., from monosaccharides to branched polysaccharides, including glycosylphosphatidylinositol, and glycosaminoglycans. The overregulated RNAs are mainly related to cell cycle, plasticity, growth regulation, invasion, metastasis, survival, proliferation, microenvironmental remodeling, and epigenetic, microbial, and immunological modulation (Table S4).

### 2.2. Transcriptomic Analysis of Differentially Overexpressed Genes (DEGs) Related to Extracellular Exosomes in Gut and Lung Tumoral- and Inflammatory-Related Tissues

In our previous cancer transcriptomic analysis, we compared the expression patterns of lung and gut normal and tumoral-related tissue [26]. LC analysis identified 417 common overregulated genes, of which 76 are related to extracellular exosomes; GC analysis identified 195 common overregulated genes, of which 47 are related to extracellular exosomes; and CC datasets analysis identified 474 common overregulated genes, of which 74 are related to extracellular exosomes [26], all according to DAVID annotation analysis (Table S5). However, LC shares 73 DEGs with LCEXO, 21 DEGs with GCEXO, and 2 with CCEXO; GC shares 75 DEGs with LCEXO, 10 DEGs with GCEXO, and 4 with CCEXO; and CC shares 104 DEGs with LCEXO, 21 DEGs with GCEXO, and 13 with CCEXO. DAVID’s annotation analysis previously identified 76 LC-common DEGs, and blood cancer exosome analysis included 67 additional LC-common DEGs that might be involved with exosomes. The 143 LC DEGs related to exosomes are involved with ATP binding, nucleotide binding, RNA binding, cell division and migration, cell cycle, and cell surface. The LC DEGs related to exosomes are also involved with epigenetic reprogramming processes like acetylation, phosphoprotein, Ubl conjugation, hydroxylation, and methylation. The LC DEGs related to exosomes include several membrane receptors (SLC1A4, SLC2A1, SLC6A8, SLC7A5, NETO2, TNFRSF21, PTPRF, SDC1, ITGB4, LRRC15, WNT5A, LSR) that might be expressed in LC cells after being transported by exosomes, which have been involved with viral and bacterial interactions, and the activation of signaling pathways related to metabolism, aminoacyl-tRNA biosynthesis, HIF-1, biosynthesis of amino acids, glycolysis, and gluconeogenesis during tumorigenesis.

DAVID’s annotation analysis previously identified 47 GC common DEGs, and blood cancer exosomes analysis included 65 additional GC common DEGs that might be involved with exosomes. The 112 GC DEGs related to exosomes are involved in integrin binding, angiogenesis, heparin binding, protease binding, cellular response to amino acid stimulus, gene expression, cell junction, proliferation and migration, membrane raft, apoptosis, symbiont entry into host cell, response to hypoxia, cytokine activity, host cell receptor for virus entry, immune and inflammatory response, and receptor internalization. The GC DEGs related to exosomes are also involved in epigenetic reprogramming processes, like glycoprotein and hydroxylation. The GC DEGs related to exosomes include several membrane receptors (CD86, TRAV25, ICAM1, CD44, ITGA1, ITGB5, CDH11, OSMR, THBS1/2/4, CLDN1, EDNRA, TNFRSF10B, TNFRSF12A, FCER1G, NOTCH3, CD9, IFITM2/3) that might be expressed in GC cells after being transported by exosomes, which have been involved in the interaction with microorganisms like Gram-negative bacterium, HSV1, HPV, entamoeba histolytica, *salmonella*, HVTL-I, HCV, and EBV, and the activation of signaling pathways related to ECM–receptor interaction, focal adhesion, PI3K-Akt, HPV infection, protein digestion and absorption, amoebiasis, integrin-mediated, proteoglycans in cancer, and phagosome.

DAVID’s annotation analysis previously identified 74 CC common DEGs, and blood cancer exosomes analysis includes 99 additional CC common DEGs that might be involved with exosome function. The 173 CC DEGs related to exosomes are involved in cell surface, adhesion, division, and junction, vesicles, host–virus interaction, integrin binding, RNA binding, apoptosis, membrane raft, angiogenesis, virus receptor activity, and symbiont entry into host cell. The CC DEGs related to exosomes are involved in epigenetic reprogramming processes like acetylation, Ubl conjugation, and phosphoprotein. The CC DEGs related to exosomes include several membrane receptors (ADGRG1, CD47, CDH11, CD44, CD81, CLDN1, EDNRA, FCGR3B, ICAM1, SLC3A2, MET, SDC3, SLC1A4, SLC7A5) that might be expressed in CC cells after being transported by exosomes, which have been involved in the interaction with microorganisms like toxoplasma, severe acute respiratory syndrome coronavirus (SARS-CoV2), HCV, HSV1, entamoeba histolytica, *Staphylococcus aureus*, malaria, EBV, and HPV, and the activation of signaling pathways related to focal adhesion, ECM–receptor interaction, proteoglycans in cancer, HCV, amoebiasis, EBV infection, TNF, relaxin, HPV infection, and protein digestion and absorption.

In our inflammatory transcriptomic analysis, we compared the expression patterns of lung and gut normal and swollen related tissue [25]. PAH analysis identified 250 common upregulated genes, of which 54 are related to extracellular exosomes; CD analysis identified 435 common upregulated DEGs, of which 61 are related to extracellular exosomes; and UC identified 522 common upregulated DEGs, of which 73 are related to extracellular exosomes [25], all according to DAVID annotation analysis (Table S5). However, PAH shares 51 DEGs with LCEXO, 14 DEGs with GCEXO, and 1 DEG with CCEXO; CD shares 36 DEGs with LCEXO, 17 DEGs with GCEXO, and 2 DEGs with CCEXO; and UC shares 51 DEGs with LCEXO, 25 DEGs with GCEXO, and 4 DEGs with CCEXO. DAVID’s annotation analysis previously identified 54 PAH common DEGs, and blood cancer exosomes analysis includes 42 additional PAH common DEGs that might be involved with exosomes. The 96 PAH DEGs related to exosomes are involved with protein binding, calcium ion binding, plasma membrane, collagen-containing extracellular matrix, retromer complex binding, membrane raft, cytoplasmic vesicle, phagocytic vesicle, negative regulation of cell population proliferation, angiogenesis, symbiont entry into host cell, endosome, lysosome, trans-Golgi network membrane, host–virus interaction, and protein transport. The PAH DEGs related to exosomes are also involved with epigenetic reprogramming processes like phosphoprotein, acetylation, and glycoprotein. The PAH DEGs related to exosomes include several membrane receptors (S1PR1, PECAM1, PSEN2, MYADM, EFNB1, EFHD2, NOTCH4) that might be expressed in PAH cells after being delivered by exosomes, which have been involved in the interaction with microorganisms like *salmonella*, legionella, HPV, *Escherichia coli*, and *Staphylococcus aureus*, and the activation of signaling pathways like Notch, efferocytosis, coronavirus disease (COVID-19), phagosome, MAPK, HCV, HPV, and *salmonella* infection, B cell receptor, and autophagy.

DAVID’s annotation analysis previously identified 61 CD common DEGs, and blood cancer exosomes analysis includes 39 additional CD common DEGs that might be involved with exosomes’ function. The 100 CD DEGs related to exosomes are involved with symport, plasma membrane, mitochondrion, cytoplasm, endosome, identical protein binding, transit peptide, phagosome, viral budding, and protein transport. The CD DEGs related to exosomes are also involved in epigenetic reprogramming processes, like lyase, phosphoprotein, and protein phosphorylation. The CD DEGs related to exosomes include several membrane receptors that might be expressed in CD cells (P2RY1, ABCB1, CHP1, PTPRF, FZD5, LPAR5, SFXN1) after being delivered by exosomes, which might be involved in the interaction with microorganisms, and the regulation of citrate cycle (TCA cycle), metabolic pathways, and pathways in cancer. DAVID’s annotation analysis previously identified 90 UC common DEGs, and the blood cancer exosomes analysis includes 60 additional UC common DEGs that might be involved with exosomes. The 150 UC DEGs related to exosomes are involved in the function of the plasma membrane, cytosol, cytoplasm, mitochondrion, endosome, nuclear envelope, and peroxisomal matrix. UC DEGs are also involved in symport, focal adhesion, cell junction, cell projection, and transport across the blood–brain barrier. The UC DEGs related to exosomes include several membrane receptors that might be expressed in UD cells (P2RX4, P2RY1, ABCB1, FCGRT, CHP1, PTPRF, FZD5, LPAR5) after being delivered by exosomes, which might be involved in the interaction with microorganisms and the regulation of multiple metabolic pathways.

### 2.3. Non-Coding RNA and mRNA Transcriptional Regulators Related to Gut and Lung Extracellular Exosome Function in Tumoral and Inflammatory Diseases

Between the overregulated LCEXO RNAs, there are 91 non-coding RNAs and 118 mRNAs transcriptional regulators (Table S1). Annotation analysis of LCEXO transcription regulators identified 72 TFs related to the positive regulation and 68 TFs related to the negative regulation of transcription by RNA polymerase II; 44 TFs are known to form transcription regulator complexes, 9 TFs are known to form transcription repressor complexes, and 22 TFs have transcription coregulator activity. LCEXO TFs are also related to regulation of immune and inflammatory responses; cell differentiation, activation, and development; miRNA transcription; apoptotic process; somatic stem cell population maintenance; stem cell differentiation; angiogenesis; proto-oncogenesis; signaling pathways like HTLV-I, HSV1, and EBV infection; type II interferon-mediated; transcriptional misregulation in cancer; pathways in cancer; C-type lectin receptor; and circadian rhythm, as well as epigenomic reprogramming processes through histone deacetylase binding, Ubl conjugation, phosphorylation, and acetylation. Between the overregulated GCEXO RNAs, there are 270 non-coding RNAs and 67 mRNA transcription regulators (Table S2). Annotation analysis of GCEXO transcriptional regulators identified 19 TFs related to the positive regulation and 23 TFs related to the negative regulation of transcription by RNA polymerase II; 11 TFs are known to form transcription regulator complexes, and 26 TFs are known to form protein–DNA complexes. GCEXO TFs are also related to the regulation of transmembrane signaling receptor activity and G protein-coupled receptor activity, positive regulation of epithelial cell proliferation, and the HSV1 infection signaling pathway, as well as epigenomic reprogramming processes like Ubl conjugation, protein dimerization activity, and chromatin DNA binding.

Between the overregulated CCEXO RNAs, there are 49 non-coding and 32 mRNA transcriptional regulators (Table S3). Annotation analysis of CCEXO transcription regulators identified 18 TFs related to the positive regulation and 11 TFs related to the negative regulation of transcription by RNA polymerase II; 3 TFs are known to form transcription regulator complexes and 16 TFs are known to form protein–DNA complexes. CCEXO TFs are also related to the regulation of epigenomic reprogramming processes like Ubl conjugation, HSV1 infection signaling pathway, defense response to tumor cells, DNA replication, cell differentiation, and the positive regulation of miRNA transcription. In blood exosomes related to gut and LC patients, there are coding transcriptional regulators already identified in our previous transcriptomic analyses [25,26].

**Figure 2:**
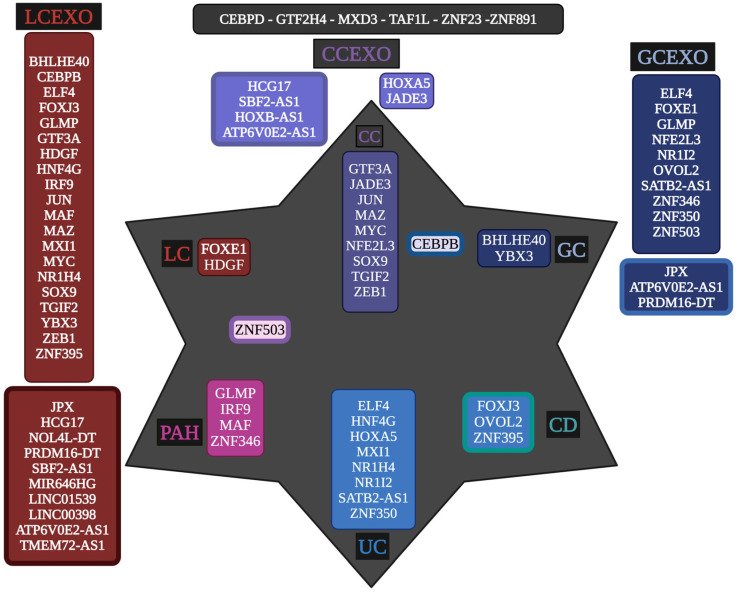
Unique and common transcriptional regulators between peripheral blood exosomes of lung cancer (LCEXO), gastric cancer (GCEXO), and colon cancer (CCEXO) patients, with the transcriptional regulatory networks (TRNs) of inflammatory- (PAH, UC, and CD) and tumoral- (LC, GC, and CC) related tissue. Created with BioRender (version 2025).

Moreover, LCEXO has twenty TFs, GCEXO has nine TFs and one lncRNA, and CCEXO has two TFs involved in inflammatory and tumoral processes (Figure 2). Then, various regulatory RNAs transported by cancer exosomes through the circulating blood might directly participate in the formation of inflammatory and tumoral TRNs that unlock phenotypic plasticity for the acquisition of the hallmarks of cancer. The TFs transported by exosomes can regulate the expression of DEGs related to the control of cellular processes, signaling pathways, and epigenetic reprogramming in gut and lung tumoral (Table 1 and Tables S1–S3) and inflammatory diseases (Table 2 and Table S6). The whole transcriptome meta-analysis highlighted 23 lncRNAs and 6 TFs (Table S4). LNCSEA 2.0 annotation analysis highlights the importance of 11 lncRNAs in gene expression regulation of lung and gut cancer cells, survival, and genomic instability (Figure 2).

### 2.4. Transcriptional Regulatory Networks (TRNs) Related to Gut and Lung Extracellular Exosomes

**Figure 3:**
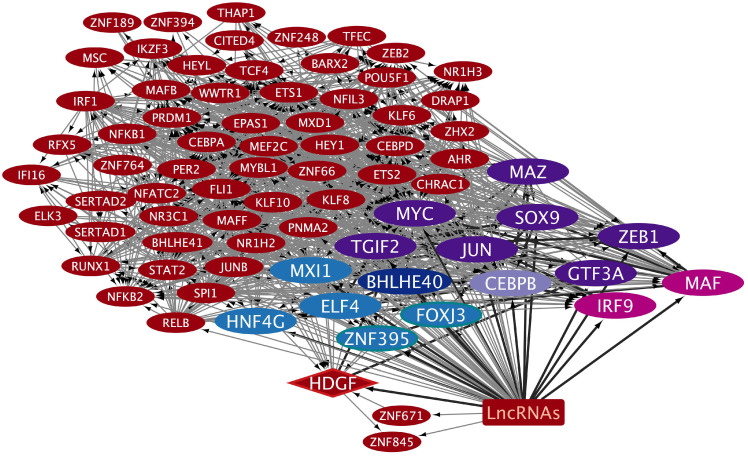
cncTRN of lung cancer (LC) serum exosomes. The TFs only related to LCEXO are in dark red ellipses, and the lncRNAs related to LCEXO are summed up in a dark red square. The LCEXO TF related to LC TRN is in a dark red diamond, to GC TRN in a dark blue ellipse, and to GC and CC TRN in a lilac ellipse. The LCEXO TFs related to CC TRN are in dark purple ellipses, to PAH TRN in pink ellipses, to UC in light blue ellipses, and to CD and UC in light blue ellipses with green borders. Created with Cytoscape Version 3.10.3.

The TRNs were constructed to analyze how exosomes might participate in the complex regulatory patterns established during inflammatory processes that induce tumorigenesis, along with cancer progression in the oral–gut–lung axis. The cncTRN analysis established predictive regulatory interactions between long non-coding RNAs and coding mRNAs (TFs) as transcriptional regulators present in whole blood serum or plasma exosomes, which might come from microbiota-, inflammatory-, and tumoral-related cells. The LCEXO cncTRN analysis highlights the interaction of 81 LCEXO LncRNA with 76 LCEXO TFs (Figure 3), 86 LCEXO TFs that might be able to regulate the expression of the 24 TFs forming the PAH TRN, and 81 LCEXO TFs that might be able to regulate the expression of 18 TFs forming the LC TRN, except for FOXE1, which could be regulated by GCEXO TF NOTO or CCEXO TF KLF8 (Table S1). LCEXO cncTRN highlights all possible RNA–RNA interactions between the transcriptional regulators from inflammatory diseases and tumoral diseases TRNs within the whole network of LCEXO TFs, which participate in gene expression regulation of PAH, CD, and UC (Table 1), as well as LC, GC, and CC (Table 2).

**Figure 4:**
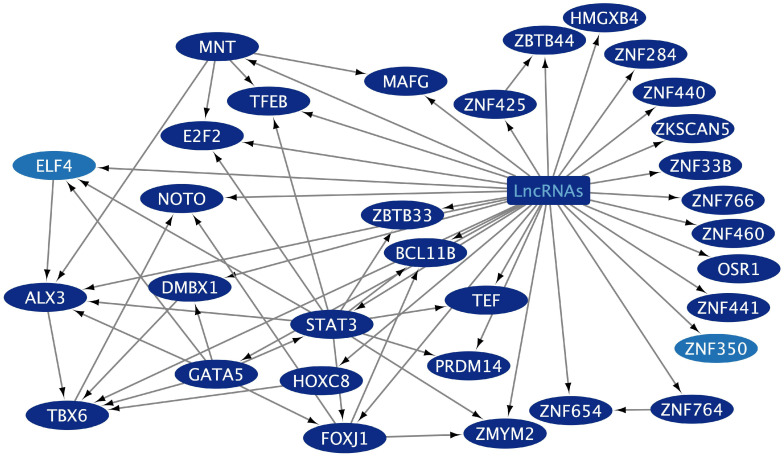
cncTRN of gastric cancer (GC) blood exosomes. The TFs only related to GCEXO are in dark blue ellipses, and the lncRNAs related to GCEXO are in a dark blue square. The GCEXO TFs related to UC are in a light blue ellipsis. Created with Cytoscape Version 3.10.3.

**Figure 5:**
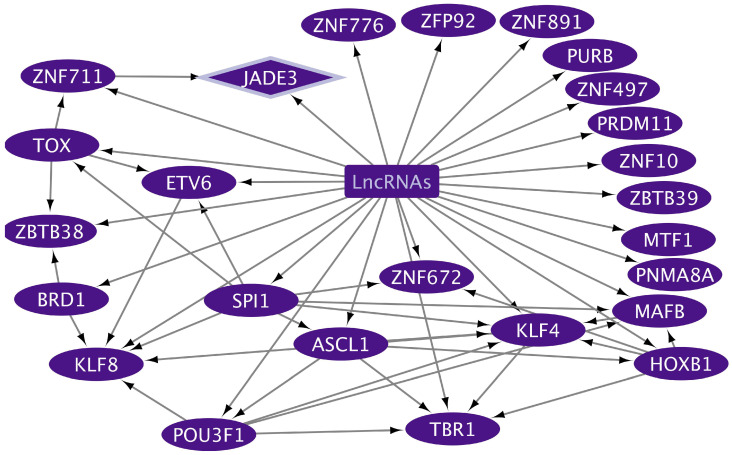
cncTRN of colon cancer (CC) plasma exosomes. The TFs only related to CC are in dark purple ellipses, and the lncRNAs related to CCEXO are in a dark purple square. CCEXO TF related to CC TRN is in a dark purple diamond. Created with Cytoscape Version 3.10.3.

The GCEXO cncTRN analysis highlights the interaction of 187 GCEXO LncRNAs with 32 GCEXO TFs (Figure 4); 55 GCEXO TFs that might be able to regulate the expression of 30 TFs forming the CD TRN, except for UBP1, which could be regulated by nine LCEXO TFs; and 54 GCEXO TFs that might be able to regulate the expression of all 11 TFs forming the GC TRN (Table S2). GCEXO cncTRN highlights all possible RNA–RNA interactions between two transcriptional regulators, also from inflammatory diseases TRNs within the whole network of GCEXO TFs, which participate in gene expression regulation of UC (Table 1). The CCEXO cncTRN analysis highlights the interaction of 46 CCEXO LncRNA with 25 CCEXO TFs (Figure 5); 140 CCEXO TFs that might be able to regulate the expression of 12 TFs forming the CD TRN and 41 TFs forming the UC TRN, except for 7 TFs, which could be regulated by 27 LCEXO TFs and 13 GCEXOTFs; and 24 CCEXO TFs that might be able to regulate the expression of 33 TFs forming the CC TRN, except for 4 TFs, which could be regulated by 27 LCEXO TFs and 8 GCEXOTFs (Table S3). CCEXO cncTRN highlights all possible RNA–RNA interactions between one transcriptional regulator from CCEXO and CC TRNs within the whole network of CCEXO TFs, which participate in gene expression regulation of CC (Table 2).

## 3. Discussion

Exosomes transport, through the blood stream, RNAs of membrane receptors related to the interaction of microbiota with gut and lung cells and exosomes regulating inflammatory and tumoral processes, and non-coding (RNAs) and coding (mRNAs) transcriptional regulators fitting into a cancer exosome cncTRNs, capable of forming coregulatory complexes through RNA–RNA and RNA–protein interactions to control the expression of TFs that belong to inflammatory and tumoral TRNs in charge of regulating other DEGs also involved in inflammatory processes and tumorigenesis in the oral–gut–lung axis.

### 3.1. Membrane Receptors of Gut–Lung Tumoral and Inflammatory Cells Transported by Exosomes for Host–Microbiota Interaction and Signaling Pathways Regulation

**Figure 6:**
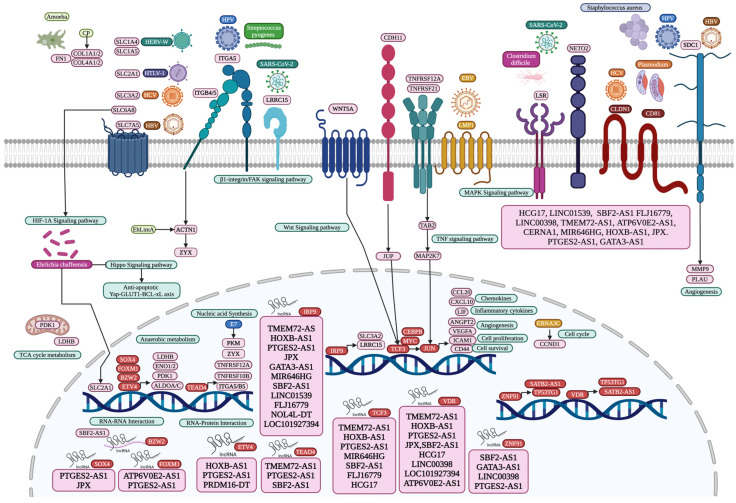
Microbiome interaction with membrane receptors of lung and gut cells activating signaling pathways involved in transcriptional regulation during tumorigenesis and inflammation, with all coding mRNAs and non-coding RNAs transported by circulating exosomes of the oral–gut–lung axis. Created with BioRender (version 2025).

Exosomes transport, through the bloodstream, mRNAs that codify receptors related to the interaction of microbiota with LC, GC, and CC cells, as well as with PAH, CD, and UC cells. The selection and relevance of these receptors is strongly supported by our previous transcriptomic analyses, which demonstrated consistent overexpression in most of the datasets evaluated in gut and lung cancer, along with inflammatory-related diseases. This evidence is complemented by experimental validation documented in the scientific literature; referenced articles confirm the physical and functional interaction between microorganisms and these receptors, reinforcing their potential role in the pathophysiology of these diseases. SLC transporters are involved in cellular energy metabolism (glucose, creatine) and protein synthesis (amino acids), and interact with bacteria, viruses, and parasites, which are crucial in health and disease [27]. SLC proteins are a group of membrane cell and organelle transporters that control the uptake and efflux of various neutral, basic, or acidic amino acids in a Na^+^-dependent or independent way [28]. SLC1A4, SLC2A1, SLC3A2, SLC6A8, and SLC7A5 are transported as mRNAs by lung- and colon cancer-related exosomes (Figure 6). SLC1A4 (ASCT1) is part of the alanine serine cysteine system, responsible for exchanging essential (threonine); non-essential (alanine, serine, and cysteine); and conditionally essential (glutamine) amino acids, making its overexpression a powerful prognostic biomarker related to cancer cell cycle progression, metabolism, proliferation, migration, anti-apoptosis, inflammation, and immunity [29,30]. The envelope glycoprotein of human endogenous retrovirus type W (HERV-W) can use SLC1A4 and SLC1A5 as receptors for cell entry [31]. SLC2A1 (GLUT1) is related to epithelial–mesenchymal transition, glycolysis, hypoxia, cell-cycle regulation, and DNA repair [32]. SLC2A1 is overregulated in LC, functioning as an HTLV-1 receptor and the regulation of aneuploidy [26]. The receptor-binding domains of the HTLV-I and -II envelope glycoproteins interact directly with SLC2A1, increasing its expression, which improves virus susceptibility [33]. *Ehrlichia chaffeensis* is an intracellular bacterium that upregulates SLC2A1 expression in infected host cells to have sufficient glucose for its own metabolism and to deactivate the Hippo pathway involved in the anti-apoptotic Yap-GLUT1-BCL-xL axis [34]. SLC2A1 is a direct receptor of viruses, while bacteria and parasites control its expression to ensure the required quantity of glucose for their own survival and replication [35]. SLC3A2 acts as a metabolic switch on lung adenocarcinoma cells to induce macrophage phenotypic reprogramming through arachidonic acid [36]. SLC3A2 is specifically required for the entry of HCV according to RNA interference technology; SLC3A2/SLC7A5 heterodimer mediates HCV entry into host cells, while HCV upregulates SLC3A2 mRNA and protein expression levels through NS3/4A-mediated oxidative stress, and increases L-leucine transport levels, leading to the activation of the mTORC1 signaling pathway [37]. There is a direct interaction between *Plasmodium vivax* parasitic ligand PvRBP2a and SLC3A2 for host cell invasion [38]. SLC6A8 (CRT1) is related to cancer patients’ survival, immune checkpoint genes, and tumor-infiltrating immune cell abundances, and is relatively accurate in identifying possible cancer patients; it contains mutations, amplifications, and deletions that might be involved in tumorigenesis through carbon metabolism and the HIF-1 signaling pathway [39]. SLC7A5 (LAT1) may be related to proliferation, migration, immunosuppressive tumor microenvironment, and the low response of immunotherapy [40]. SLC7A5 is also overregulated in LC that might also function as an HBV receptor, regulating the tumor immune microenvironment [26].

Integrins are heterodimer transmembrane proteins, with α subunits that regulate integrin activation, and β subunits that mediate intercellular and cell-ECM interactions [41]. Integrin beta 4 (ITGB4) forms a critical link between the extracellular matrix and the cell interior by interacting with focal adhesion via paxillin, and its presence in tumor-derived exosomes facilitates the creation of a microenvironment that promotes LC [42]. SLC3A2 can associate with ITGB4 in LC to regulate integrin signaling, cell survival, and cell migration, and in turn, ITGB4 might be related to leucine-rich repeat-containing protein 15 (LRRC15), which may promote cancer metastasis by affecting cell–cell and cell–matrix interactions (Figure 6), and by activating the β1-integrin/FAK signaling pathway [43]. LRRC15 is a SARS-CoV-2 spike-binding receptor that may regulate viral load, antiviral and antifibrotic transcriptional programs during COVID-19 infection [44]. Moreover, HCV may upregulate SLC3A2, contributing to HCV-mediated pathogenesis, in association with ITGB4 during tumorigenesis [26]. Integrin subunit alpha 5 (ITGA5) belongs to the integrin alpha chain family of extracellular matrix receptors, is vital for promoting cancer cell invasion and metastasis, is significantly associated with tumor purity and immune infiltration levels of different immune cells in gastrointestinal tumors [45], and is positively correlated with lymph node metastasis and the TNM stage of GC, shorter overall survival, and disease-free survival [46]. Integrin subunit beta 5 (ITGB5) acts as a scaffold to enhance GC metastasis by promoting the endosomal recycling of TGFβ receptors, sustaining TGFβ signaling, and promoting EMT [47]. ITGA5 and ITGB5 may form a heterodimer in GC that promotes the integrin-mediated signaling pathway, viral entry into the host, HPV infection, phagosome, focal adhesion, ECM–receptor interaction, and the cell junction [26]. ITGA5 participates in the positive regulation of cell migration, along with tight junction protein claudin-1 (CLDN1), which is required for efficient HCV virion accumulation at the tight junction from the basolateral membrane, along with viral envelope glycoproteins E1 and E2 [48], and tetraspanin protein CD81 [49]. CLDN1 is correlated with tumor infiltration, metastasis, enhanced anoikis resistance, poor survival, cell aggregation, cell migration, and colonization [50]. CD81 is involved in *parasitophorous* vacuole membrane formation and organization during *Plasmodium* sporozoite invasion [51]. ITGB4 directly interacts with the E glycoprotein of *Zika* virus, mediating its attachment, entry, and infection of host cells [52]. Overexpression of ITGB5 significantly enhances adhesion of intestinal epithelial cells to enterotoxigenic *Escherichia coli* [53]. The integrin formed by ITGA1 and ITGB1 binds to the M49 strain of *Streptococcus pyogenes* to invade cells via its plasminogen/plasmin-binding M protein [54], as well as the non-structural protein 4 (NSP4) of rotavirus facilitating its enterotoxin function [55], and the adherence and colonization protein of group B Streptococcus to promote its internalization in epithelial cells [56].

Syndecan-1 (SDC1) is a member of the transmembrane heparan sulfate proteoglycan family, essential for intestinal barrier function, epithelial junction formation and maintenance, and alleviates inflammation through a network including nuclear factor (NF)-κB and microRNAs [57]. SDC1 regulates epithelial plasticity and coordinates lung fibrosis programs, altering alveolar type II cell phenotypes through the activation of profibrotic pathways like TGF-β and Wnt, controlling the packaging of several antifibrotic miRNAs into EVs that have wide outcomes over those fibrogenic signaling networks [58]. SDC1 might be related to HPV and HBV internalization into epithelial cells in LC (Figure 6) [26]. SDC1 promotes Streptococcus pneumoniae epithelial cells’ extracellular matrix attachment through its PavA protein, establishing a niche for cell growth and infection [59]. SDC1 promotes *Listeria monocytogenes* infection, inhibiting the formation of neutrophil extracellular traps that would induce its clearance, while *L. monocytogenes* induces SDC1 shedding to disrupt this host defense [60]. SDC1 shedding can also be induced by *Pseudomonas aeruginosa*, as the soluble ectodomains enhance its virulence by inhibiting innate immune defense [61]. CD9 is a tetraspanin able to synchronize SDC1, fibronectin, and β1 integrins to allow efficient *Staphylococcus aureus* adhesion to several types of host cells [62], and infection by interfering with the capacity of neutrophils to kill *Staphylococcus aureus* [63], and in turn, *Staphylococcus aureus* induces SDC1 shedding in vitro through α- and β-toxins [64]. HCV viremia induces an increase in CD86 expression and the imbalance of cell maturation, representing a mechanism of evasion of the immune system [65]. The cluster-of-differentiation gene (CD44) is a transmembrane receptor related to epithelial cell proliferation induced by *H. pylori,* which leads to CD44 expression during inflammation and disease progression [66]. *Streptococcus pyogenes* hyaluronic acid capsule binds to the CD44 receptor on host cells, facilitating adhesion and potentially leading to invasion, leading to cytoskeletal rearrangements in human epithelial cells, which disrupts intracellular junctions and allows the dissemination of streptococci into deeper, underlying sterile tissue [67]. CD44 is a differentially expressed protein in *Leishmania*-infected host cells [68]. Syndecan-3 (SDC3) or N-syndecan is a transmembrane heparan sulfate proteoglycan related to development, cell adhesion, migration, and shape organization. It is a co-receptor of growth factors, cytokines, chemokines, and other signaling molecules, modulating various signaling pathways [69]. SDC3 expression is related to tumor-associated macrophages, cancer, and endothelial cells, and hypoxia in the tumor microenvironment [69].

Wnt Family Member 5A (WNT5A) is a glycoprotein of the β-catenin-independent branch, acting as a signaling molecule that plays a crucial role in various developmental processes, which is involved in processes like cell migration, adhesion, and polarity during both normal development and disease, including cancer [70]. WNT5A is related to LC cell proliferation, migration, and the epithelial–mesenchymal transition [71]. Cigarette smoke exposure amplifies WNT5A expression at both mRNA and protein levels in human tissues, favoring chronic inflammatory conditions and cancer development [72]. *Helicobacter pylori* infection can activate the WNT/β-catenin signaling pathway, inducing WNT5A expression, inflammation, and gastric cancer, as well as gastric stem cell generation and proliferation [73]. *Porphyromonas gingivalis* infection is related to periodontitis and can also modulate the WNT signaling pathway, including WNT5A, which increases the activity of the β-catenin-dependent TCF/LEF promoter in epithelial cells (Figure 6), which may contribute to a proliferative phenotype and modulate the inflammatory processes [74].

Intercellular adhesion molecule 1 (ICAM-1) is a surface glycoprotein, a transmembrane protein in the immunoglobulin superfamily, and an adhesion receptor involved in transendothelial migration of lymphocytes and leukocyte recruitment from circulation to sites of inflammation, transduction outside-in-signaling, and epithelial injury-resolution responses, as well as immune cell effector function in inflammation and tumorigenesis [75]. ICAM-1 increases the efficiency of HTLV-1 infection [26]. ICAM-1 is upregulated in epithelial cells of large and small airways of chronic airflow limitation patients, crucial for the infection of around 60% of human rhinoviruses, and non-typeable *Haemophilus influenzae* [76]. Plasmodium falciparum-infected erythrocytes express PfEMP1 on the surface to bind to ICAM-1 of endothelial cells and other cells, leading to its sequestration in blood vessels and tissue, microvascular obstruction, and inflammation [77]. ICAM-1 and CD44 are massively recruited to bacterial adhesion sites during *Neisseria meningitidis* infection, together with the ERM proteins (ezrin), to regulate host inflammatory response by blocking the transendothelial migration of leukocytes [78].

Neuropilin and tolloid-like 2 (NETO2) is a common overexpressed gene LC, which may also be related to HTLV-1 infection [26], regulating aneuploidy, metastasis, cell proliferation, apoptosis, tumor growth, and ERK phosphorylation [79]. NETO2 and lipolysis-stimulated lipoprotein receptor (LSR) may also activate SARS-CoV-2 S proteins and increase the viral infection of LC cells, while LSR is related to *Clostridium difficile* cell binding (Figure 6), to regulate cell proliferation, invasion, and migration via MAPK signaling [26]. Microbial antigens may be presented via a major histocompatibility complex to T cells expressing specific T Cell Receptor Alpha Variable 25 (TRAV25) regions and initiate an immune response [80]. Thrombospondin-1 (THBS1) is a secreted matricellular glycoprotein that modulates cell behavior by interacting with components of the extracellular matrix and with several cell surface receptors [81]. Thrombospondin has a conserved structural repeat motif implicated in receptor binding during *Plasmodium falciparum* cell invasion [82]. *Borrelia burgdorferi* is a spirochete able to take over multiple human cells’ extracellular matrices despite strong host immune responses [83]. CD47 is an integrin-associated protein able to suppress phagocytosis, which interacts with other innate immune receptors like TLR2, related to intracellular *Porphyromonas gingivalis* survival in macrophages depending on the bacterial major fimbria, and causes the increased expression and secretion of the CD47 ligand THBS1 following *Porphyromonas gingivalis* infection, which protects periodontitis-associated bacterial species from neutrophil bactericidal activity, helping bacteria survive in an inflamed environment and cause dysbiosis [84]. CD47 is overexpressed in cells infected by pathogens, including SARS-CoV-2, induced by endosomal and cytosolic pathogen recognition receptors in HCV patients, and is related to innate adaptive immune responses during infections with lymphocytic choriomeningitis virus and *Mycobacterium tuberculosis* [85]. c-Met (Hepatocyte Growth Factor Receptor) (MET) is also related to *Mycobacterium tuberculosis* infection, activating macrophage and cytokine production, cell survival, antigen presentation, and microbicidal activities, and triggering host type I interferon signaling to regulate IL-1β production in human macrophages [86]. *Listeria monocytogenes* binds to MET through its surface protein InlB to promote signaling pathways for the internalization of bacteria into non-phagocytic cells, including MET tyrosine phosphorylation [87]. Fc Gamma Receptor IIIb (FCGR3B) is a low-affinity receptor for the Fc region of IgG, facilitating the interaction between host cells with bacteria and viruses that have been coated with IgG antibodies, regulating immune responses and leading to phagocytosis, degranulation, and antibody-dependent cellular cytotoxicity [88]. HPV capsid proteins L1 and L2 may induce virus internalization, probably through the attachment to neurogenic locus notch homolog protein 3 (NOTCH3) or EPH receptor B2 (EPHB2) in gastric cancer. Moreover, EBV might use G protein-coupled receptor (GPCR) signaling, like EDNRA and ADGRG1, which are common DEGs in colon cancer [26]. CD81 is a coreceptor that efficiently mediates HCV attachment and entry into target cells through its envelope glycoproteins E1/E2, which are pseudotype retroviral particles [89]. Several membrane receptors identified as key deregulated genes in cancer and inflammatory diseases of the gut and the lung transported by exosomes have no direct experimental evidence related to their interaction with microorganisms; however, most of them are related to several cellular processes like inflammatory and immune responses mediated by bacteria and parasites, influencing bacterial adhesion or transmigration, and signaling pathways to facilitate their dissemination or evade immune clearance.

### 3.2. Transcriptional Regulators of Gut–Lung Tumoral and Inflammatory Cells Transported by Exosomes

Exosomes also transport, through the bloodstream, RNAs that codify transcriptional regulators that belong to inflammatory and tumoral TRNs. Forkhead box protein E1 (FOXE1) and Hepatoma-derived growth factor (HDGF) are TFs of the LC regulatory network [26], transported by LCEXO and GCEXO (Figure 2). FOXE1 is overexpressed in non-small cell lung cancer (NSCLC), playing a role in the downregulation of autophagy markers and the upregulation of matrix metalloproteinases pathways [90]. Hepatoma-derived growth factor (HDGF) is also overexpressed in NSCLC, promoting proliferation, migration, and invasion, and is correlated with tumor relapses and poor survival rates [91]. Basic Helix-Loop-Helix Family Member e40 (BHLHE40), CCAAT/Enhancer Binding Protein Beta (CEBPB), and Y-box Binding Protein 3 (YBX3) are TFs of the GC regulatory network [26], transported by LCEXO. BHLHE40 is overexpressed in GC cells and tissues, activating growth, mobility, and glycolysis [92,93]. CEBPB is overexpressed in GC and is associated with cell differentiation, proliferation, and tumorigenesis [94]. YBX3 is overexpressed in GC cells and is related to cell proliferation and metastasis [95], and when it is transported by exosomes may promote angiogenesis in vascular endothelial cells by enhancing the expression of angiogenic factors [96]. CEBPB, General Transcription Factor IIIA (GTF3A), JUN, Myc-associated zinc finger protein (MAZ), proto-oncogene (MYC), SOX9, Homeobox protein transforming growth factor beta-induced factor 2 (TGIF2), and Zinc finger E-box-binding homeobox 1 (ZEB1) are CC oncogenes related to cell proliferation, invasion, and metastasis of colon cancer [97,98,99,100,101,102,103,104]. Jade family PHD finger 3 (JADE3) is related to cell proliferation and apoptosis and increases cancer stem cell-like properties along with SOX9 [105,106]. NFE2L3 may modulate the tumor microenvironment and control colon cancer cell growth [107,108]. ZNF503 promotes migration, invasion, and EMT [109]. Moreover, the annotation analysis of the targets of LC, GC, and CC TFs according to the GRN shows that they are involved in multiple cellular processes, signaling pathways, and epigenetic reprogramming, suggesting that they could potentially become gut–lung cancer biomarkers and therapeutic targets (Table 1).

Interferon Regulatory Factor 9 (IRF9) is upregulated in pulmonary artery smooth muscle cells in PAH and is related to mitochondrial dysfunction [110]; in LC, it is associated with reduced survival, promoting proliferation, migration, and tumor growth [111]. Glycosylation-dependent cell adhesion molecule 1-like protein (GLMP) is a mucin-like endothelial glycoprotein involved in cell adhesion and gene transcription [112]. V-maf avian musculoaponeurotic fibrosarcoma oncogene homolog (MAF) is a member of the activator protein-1 family involved in cell cycle, proliferation, oxidative stress, and inflammation [113]. Zinc Finger Protein 346 (ZNF346) plays a role in metabolism, autophagy, apoptosis, immune responses, stemness maintenance, and differentiation [114]. Zinc Finger Protein 503 (ZNF503) promotes migration, invasion, and the EMT process through regulating GATA3 expression [115]. Forkhead box protein J3 (FOXJ3) expression is positively correlated with stomach adenocarcinoma and involved in histone modification, the TGF-β signaling pathway, and chromatin organization [115]. Ovo Like Zinc Finger 2 (OVOL2) is involved in embryo development and adult tissue homeostasis and regulates tumor growth and metastasis, promoting EMT while inhibiting autophagy; alters the expression of inflammation-related factors; and organizes a regulatory network to control energy homeostasis [116]. Zinc Finger Protein 395 (ZNF395) contributes to hypoxia-associated inflammation by inducing the expression of proinflammatory cytokines [117]. Transcription factor E74, like ETS transcription factor 4 (ELF4), is dysregulated by posttranslational modifications, gene fusions, and complex signaling crosstalk, exhibiting both tumor-suppressing and tumor-promoting effects. Specifically, it plays a key role in cancer metastasis, proliferation, and modulation of the tumor microenvironment [118]. Hepatocyte Nuclear Factor 4-gamma (HNF4G) is an orphan nuclear receptor superfamily involved in intestinal epithelial cell differentiation and function, which might promote tumor growth and invasion by inhibiting apoptosis [119]. Homeobox protein Hox-A5 (HOXA5) regulates proliferation, differentiation, invasion, apoptosis, cancer stem cell progression, and the immune microenvironment [120]. MAX interactor 1 (MXI1) regulates Myc, cellular growth, and differentiation [121]. Farnesoid X receptor (NR1H4) is a nuclear receptor with a role in metabolic processes, and a ligand-activated transcription factor that regulates homeostasis, lipogenesis, gluconeogenesis, ammonia detoxification, glycogen synthesis, and inflammation [122]. Pregnane X receptor (NR1I2), or steroid and xenobiotic receptor (SXR), is a nuclear receptor that regulates drug metabolism and detoxification, glucose, lipid, energy homeostasis, inflammatory response, cell proliferation, apoptosis, and cell migration [123]. Zinc Finger Protein 350 (ZNF350) regulates tumor growth, metastasis, and inflammation [124]. Moreover, the annotation analysis of the targets of PAH, CD, and UC TFs, according to the GRN, shows that they are involved in multiple cellular processes, signaling pathways, and epigenetic reprogramming, suggesting that they could potentially become gut–lung inflammatory and cancer early detection biomarkers and therapeutic targets (Table 2).

Analyzing cncTRNs within circulating exosomes helped to reveal how extracellular vesicles contribute to the interplay between microbiota, inflammation, cancer establishment, and progression across interconnected sites of the oral–gut–lung axis, where microbial translocation and systemic inflammatory and tumorigenic signaling may occur through exosomes derived from host cells or even potentially from the microbiota carrying both coding mRNA (e.g., PAH, CD, UC, LC, GC, and CC TFs) and non-coding RNA (e.g., lncRNA) transcriptional regulators transferring regulatory information between different cell types organs and systems, as well as other RNAs that codify for cell membrane receptors that may interact with microbiota capable to shape local and systemic inflammatory and immune responses to specifically influence and transform cells microenvironment. LCEXO cncTRN highlights all possible RNA–RNA interactions between the transcriptional regulators from inflammatory disease and tumoral disease TRNs within the whole network of LCEXO TFs, which participate in gene expression regulation of PAH, CD, and UC, as well as LC, GC, and CC (Figure 3). GCEXO cncTRN highlights all possible RNA–RNA interactions between two transcriptional regulators, also from inflammatory diseases’ TRNs, within the whole network of GCEXO TFs, which participate in gene expression regulation of UC (Figure 4). CCEXO cncTRN highlights all possible RNA–RNA interactions between one transcriptional regulator from CCEXO and CC TRNs within the whole network of CCEXO TFs, which participate in gene expression regulation of CC (Figure 5). LncRNAs modulate various aspects of gene regulation in a highly tissue- and context-specific manner in all cell pathways in normal or disease conditions [125]. LncRNAs can guide TFs to their target DNA sites or genes in the nucleus and modulate their activity, acting as co-activators or co-repressors of transcription, regulating TF interaction with their intrinsic target in space and time, inducing allosteric changes in the TF structure, affecting TF DNA-binding affinity and the interaction with other co-activators or co-repressors, TF stability, TF post-translational modifications, promotion or inhibition of TF degradation, TF subcellular localization, and TF availability [126]. In cancer, lncRNA can act as oncogenes or tumor suppressors, where lncRNA promoters and/or their gene bodies can regulate TF expression [127], as seen in cncTRNs of gut and lung cancer, where lncRNAs are part of a network of inflammatory and tumoral diseases as key regulators of TFs (Figure 3, Figure 4 and Figure 5). TFs can also bind to the promoter regions of lncRNA genes and regulate their transcription, which creates complex regulatory circuits and feedback loops [128]. The microbiota can also modulate lncRNA expression with downstream effects on host physiology, like promoting host metabolism [129], and the abnormal expression of lncRNAs can promote CC cell growth, proliferation, and metastasis, mediating the effects of the gut microbiome [130]. LncRNA SATB2-AS1 can promote tumor growth and metastasis and affects the tumor immune microenvironment by regulating SATB2, a known overregulated TF in UC [25]. However, common deregulated lncRNAs and other types of ncRNAs must be identified in transcriptomic studies from different populations, to clarify which are key to inflammatory and tumoral processes and therefore hold great diagnostic and therapeutic potential, especially in their TF interactions possible involvement in regulating vector–host–pathogen interactions [131].

### 3.3. The Whole Transcriptome Meta-Analysis of Lung, Gastric, and Colon Cancer Circulating Exosomes

Some of the proteins codified by the key mRNAs identified in the meta-analysis have a signal sequence, a short sequence of amino acids (typically between 15 and 30, with ∼20 being a common value) usually found at the N-terminus of a newly synthesized protein, which acts as a “targeting tag” that directs the protein to the endoplasmic reticulum (ER), where the protein enters the secretory pathway (ER → Golgi apparatus → Secretory vesicles or Plasma membrane) and it is cleaved by a signal peptidase after the protein has been correctly anchored to the cell membrane or secreted [132]. Some secreted proteins, such as growth factors, cytokines, or extracellular matrix components (e.g., Bone morphogenetic protein 2 (BMP2), ADAM metallopeptidase with thrombospondin type 1 motif 16 (ADAMTS16), Tenascin-XB (TNXB)) are released to degrade the surrounding matrix for tumor microenvironment remodeling, creating pathways for invasion and metastasis [133]. Adhesion and extracellular matrix proteins (e.g., PLXNA4, PCDHGA6, BCAN) are targeted to the membrane or externally to disrupt cell–cell and cell–matrix interactions, which are critical for the invasion process and the formation of metastatic foci [134]. Other membrane proteins, such as GPCRs (e.g., GRM6, GPR37L1, NMUR2), are highly expressed to mediate growth and survival signaling, amplify tumor proliferation signals, and prevent apoptosis [135,136].

The proteins encoded by key meta-analysis genes also contain repeated sequences that are used by tumor cells primarily for plasticity, adaptability, and immune escape, as these repeats often confer structural flexibility and a high capacity for mutation or variation [137]. ECM proteins such as TNXB and BCAN, and cytoskeletal components such as TUBB, contain modular repeats which can be overexpressed in cancer, which is a major factor driving tumor invasion and metastasis, as they change ECM physical properties and tumor cell mechanics, which facilitates tissue invasion and cell migration, as the tumor cell needs to actively degrade and remodel the surrounding matrix to move [138]. These repeated sequences in proteins can become neoantigens, marking the tumor for the immune response [139]. Repeats can be sites of massive O-glycosylation, as in GALNT16, creating a protective layer of sugars that masks cancer cells from immune surveillance, as high variability also confuses the development of targeted immunotherapies [140]. PRDM16-DT, ZNF891, and ZNF23 are often epigenetic regulators or transcription factors with zinc finger repeat domains, while ZNF proteins have DNA-binding transcription factor roles, and PRDM16-DT functions as an lncRNA that can influence gene expression through interactions with other proteins [141,142]. Variation in the number of PRDM16-DT repeats can alter how these proteins bind to DNA or other proteins, allowing tumor cells to rapidly change their gene expression profile to adapt to treatments or new microenvironments, as when they metastasize [143,144].

Regulatory complex proteins involved in transcription and transported by exosomes as RNA molecules identified in the meta-analysis may control which genes are activated or repressed to promote the malignant phenotype (Figure 2). ZNF23 and ZNF891 belong to the largest family of TFs, capable of acting as oncogenes or tumor suppressors in the development, progression, and metastasis of malignant tumors via regulating gene transcription and translation processes in different cancer types, probably associated with their specific interactome [145]. GTF2H4 and TAF1L are components of the basal transcription machinery that play key roles in regulating gene transcription in eukaryotes. General TF IIH Subunit 4 (GTF2H4) is a subunit of the DNA repair and transcription complex TFIIH and has been linked to lung cancer susceptibility, promoting partial endothelial-to-mesenchymal transition and angiogenesis under hypoxic conditions in ischemic diseases, suggesting it may play a role in promoting tumor vascularization and growth [146]. TATA-box Binding Protein Associated Factor 1 Like is a homolog of TAF1 (TAF1L), part of the general transcription factor TFIID, and acts as a transcriptional co-activator and possesses histone acetyltransferase activity, which is involved in loosening chromatin structure to facilitate transcription, promoting cell growth, proliferation, migration, and invasion and decreasing autophagy-dependent apoptosis, by upregulating proto-oncogenes like c-Myc and downregulating tumor suppressors like p53 via the Akt signaling pathway [147,148]. Max dimerization protein 3 (MXD3) is a transcription factor that plays a crucial role in cell proliferation by regulating gene transcription, and its overexpression has been associated with decreased sensitivity of cancer cell lines to several mitogen-activated protein kinase inhibitors, and increased activities of other kinase inhibitors, including Akt inhibitors, suggesting that it is an immune-oncogenic molecule and could become a biomarker for cancer detection, prognosis, and therapeutic design [149]. CCAAT/Enhancer-Binding Protein delta (CEBPD) is a TF involved in differentiation, inflammation, and the tumor-promoting microenvironment, aids hypoxia adaptation and cell proliferation, and contributes to the recruitment of blood vessels for improved nutrient supply to tumor cells and facilitated extravasation [150]. These TFs must be experimentally validated to clearly understand their function in the context of exosomes and inflammatory and tumorigenic processes.

A meta-analysis of lncRNAs showed that they are related to the eQTL, a genetic marker that determines how actively a gene is “turned on” or “turned off” [151]. LNCSEA 2.0 annotation analysis predicts that HCG17 in colon adenocarcinoma, MIR646HG, SBF2-AS1, HCG17, LINC01539, LINC00398, TMEM72-AS1, and ATP6V0E2-AS1 in lung adenocarcinoma, and in ATP6V0E2-AS1 in stomach adenocarcinoma, may be involved in the control of gene expression, suggesting the importance of exosomes in regulatory processes during tumorigenesis (Figure 2). Likewise, lncRNA expression is also frequently controlled by eQTLs that have also been selected for their role in cancer risk, suggesting their strong potential relevance to cancer immunity and treatment [152]. In lung, colon, and gastric cancer, where epithelial homeostasis is deregulated, an lncRNA whose expression is controlled by an eQTL can destabilize this homeostasis, initiating malignant transformation [153]. Hence, if an eQTL increases the expression of a particular lncRNA, this lncRNA “sponges” more miRNAs, resulting in overexpression of the functional oncogene (the target gene), promoting proliferation, and is a fundamental mechanism for the development of colon, gastric, and lung cancer [154]. LncRNAs and TFs interact in cancer to influence gene expression through multiple mechanisms: lncRNAs acting as decoys to block TFs binding; lncRNAs can form complexes with transcription factors to alter their activity, stability, localization, or ability to interact with other proteins, affecting cancer cell functions like proliferation and metastasis, crucial in cancer development and progression and are being explored as potential therapeutic targets; lncRNAs transcribed from enhancer regions can promote the formation of chromatin loops by interacting with transcription factors, bringing distant gene promoters into proximity with enhancers, activating transcription; lncRNAs can be regulated by transcription factors, creating complex feedback loops [155]. LNCSEA 2.0 annotation analysis predicts that the key TFs for lung (SOX4, FOXM1, and IRF9) and gut (TEAD4, ETV4, TCF3, ZNF91, and VDR) tumoral and inflammatory processes [25,26] may interact as proteins with key metanalysis lncRNAs, while BZW2 establishes RNA–RNA interactions with SBF2-AS1 (Figure 6). The function of these RNA–protein and RNA–RNA interactions must be thoroughly investigated experimentally to establish their importance in gene expression regulation processes at the transcriptomic level. However, it suggests the importance of exosomes in the transport of RNAs that regulate expression in inflammatory and tumor cells along the oral–gut–lung axis.

Pseudogenes are copies of functional genes that have lost their ability to code for a protein due to mutations (such as premature stop codons or deletions), and exhibit great potential as biomarkers and therapeutic targets for future cancer treatment [156]. FK506-binding protein 1A (FKBP1A) exhibits differential expression in cancer, serves as a prognostic indicator, functions as a pro-oncogenic factor that promotes cell proliferation and tumor aggressiveness, undergoes genetic alterations, and influences the tumor immune microenvironment [157]. Pseudogenes can also increase the production of proteins involved in steroid metabolism or drug resistance, such as can amplify the expression of RNA polymerase or related proteins, promoting a high growth rate and protein/ribosome synthesis [158]. Pseudogenes can be actively utilized by tumor cells for their role as regulators of expression of their functional parent gene through a mechanism known as competition for endogenous RNAs (ceRNAs), as they share binding sites for the same miRNAs, acting as a “sponge” that sequesters and traps miRNAs that would otherwise bind to and degrade the parent gene’s mRNA, which increases the stability and translation of the parent gene’s mRNA [159]. Through meta-analysis, pseudogenes have been identified as overregulated in cancer, but their specific function has not been described in any tumoral process [160]. However, the lncRNA GATA3-AS1 sponges miR-30b-5p, which in turn leads to the upregulation of the target gene Tex10, regulating tumor cell growth, viability, proliferation, invasion, apoptosis, and stemness, as well as Wnt1/β-catenin signaling [161]. The high expression of GATA3-AS1 is significantly correlated with larger tumor size, advanced TNM stage, and more lymph node metastasis, downregulating tumor suppressors [162], and its binding with MLL methyltransferase components forms a DNA-RNA hybrid (R-loop), tethering it to the gene locus, to regulate T helper cell differentiation [163]. The HOXB cluster antisense RNA 1 (HOXB-AS1) is related to cancer cell proliferation, migration, and invasiveness, which could become a biomarker for early diagnosis and prognosis [164]. HOXB-AS1 may also act as a ceRNA to promote metastasis [165], upregulating the expression of the transcription factor SOX4 by competitively binding to certain miRNAs [165], which activates downstream genes involved in the acquisition of the hallmarks of cancer [26]. JPX is involved with cancer growth, metastasis, and chemoresistance, also by acting as a competing endogenous RNA for microRNA, interacting with proteins, and regulating some specific signaling pathways [166]. GATA3-AS1, HOXB-AS1, and JPX may modulate the expression of key genes (including lineage genes and oncogenes), driving aberrant growth and differentiation.

The coding proteins in the meta-analysis also contain disulfide bonds, which are used by tumor cells to stabilize their three-dimensional structure in hostile extracellular environments, making them resistant to degradation by proteases or denaturation in variable environments, key to promoting invasion and metastasis [167]. Disulfide bonds stabilize extracellular proteins like BMP2 and TNXB by providing covalent linkages that reinforce their structure against the harsh, oxidizing environment outside the cell, crucial for their biological function, maintain their active, functional form outside the cell, even under stress, as they cannot rely on the protective, reducing environment and chaperone proteins found inside the cell [168,169]. Cancer cells can invade tissues by keeping matrix-degrading enzymes like ADAMTS16 active, and for membrane receptors to continue responding to growth signals, promoting cell migration and invasion [170]. The oxidation and reduction in disulfide bonds in ADAMTS16 enzyme and associated receptors may act as a key regulatory switch for tumor-promoting activity and drug resistance in cancer cells [171]. Tumor cells exploit and alter their redox machinery that maintains the cellular oxidation-reduction balance to control these bonds, creating an environment that favors their survival and malignancy [172].

Aberrant glycosylation is a hallmark of cancer [173]. A significant number of proteins encoded by the genes in the meta-analysis, which are secreted or membrane-anchored, are glycoproteins that can be used by tumor cells primarily for extracellular communication, adhesion, and immune escape, as well as in fundamental cancer processes like cell signaling, invasion, angiogenesis, and metastasis [174]. TNXB and brevican (BCAN) are usually heavily glycosylated [175], remodel the ECM and promote motility, creating a scaffold that facilitates tumor growth, migration, and invasion [176]. GRM6, GPR37L1, NMUR2, and ADGRV1 are G protein-coupled receptors (GPCRs) whose extracellular domains are glycosylated and are involved in growth and survival signaling, amplifying proliferation and angiogenesis signals [177,178,179]. Tumor cells often overexpress glycosyltransferases, particularly the GALNT family, which produces unique and abnormal glycan patterns primarily by initiating or altering O-glycosylation, probably supported by GALNT9-AS1, altering the function of cell surface proteins, altering critical cellular processes like adhesion, signaling, and immune evasion to promote cancer progression by Wnt/β-catenin signaling pathway via abnormal o-glycosylation of CD44 to enhance malignancy [180]. A dense, negatively charged layer of glycans can physically hide tumor-associated antigens from immune cell receptors, preventing T-cell receptors and NK cell activating receptors from binding to their target ligands on the cancer cell surface [181]. In the lectin pathway, lectin-associated serine protease 2 (MASP2) is activated with pattern-recognition molecules like mannose-binding lectin (MBL) and ficolins, which bind to carbohydrate structures, such as the mannose residues often found on the surface of pathogens or aberrantly glycosylated tumor cells [182]. Activated MASP-2 then cleaves complement components C4 and C2, which leads to the formation of the C3 convertase enzyme complex (C4b2a), initiating the complement cascade to promote inflammation, which leads to the formation of the membrane attack complex (MAC), the inhibition of the function of NK cells, and the control of their related cytokines and their ability to induce antitumor responses [183]. Glycosylation confers secreted or membrane proteins greater resistance to proteolysis and stressful conditions in the tumor microenvironment, such as hypoxia, acidity, ensuring that proteins essential for tumor survival, such as growth factors or ECM components that form metastatic niches, remain functional for longer, maintaining the cancer’s proliferative and invasive advantage, facilitating tumor progression, metastasis, and immune evasion [184].

The regulation of cell death, inflammation, and immune escape in cancer is associated with the TNF signaling pathway. TNFRSF12A (Fn14) is a receptor for the TWEAK ligand, frequently overexpressed in many cancers, associated with cell survival, migration, and metastasis [185]. TNFRSF21 (DR6) is a receptor involved in regulating the immune response that contributes to malignant survival, promoting tumor aggressiveness in lung cancer by increasing ERK/FOXM1 signaling, and cancer stem cell characteristics by promoting CD133 and CD44 mRNA expression [186]. *Epstein–Barr virus* (EBV) is closely associated with multiple human cancers, including carcinomas derived from epithelial cells, where it is induced by activating various signaling pathways, such as nuclear factor-κB (NF-κB), phosphoinositide-3-kinase/protein kinase B (PI3K/AKT), Janus kinase/signal transducer and transcription activator (JAK/STAT), mitogen-activated protein kinase (MAPK), transforming growth factor-β (TGF-β), and Wnt/β-catenin (Figure 6), which are regulated by EBV-encoded proteins and non-coding RNA [187]. EBV’s interaction with the TNF signaling pathway helps create an immunosuppressive tumor microenvironment that supports tumor growth, as LMP1 upregulate their own TNF-α production, to protect the infected cell from apoptosis, while the TNF-α they secrete can kill surrounding, uninfected cells, promoting chronic inflammation and tissue damage, and resistant of the infected cells, inducing the expression of multiple downstream targets involved in chronic inflammatory responses, like interleukins, chemokines, adhesion molecules; antigen processing and presentation proteins, all of which are involved in immune evasion, cell growth, glycolysis, angiogenesis, invasion, metastasis, and epithelial–mesenchymal transition [188]. LNCSEA 2.0 annotation analysis predicts the interaction of several lncRNAs, suggesting that they are deeply related to multiple cancer immunology signaling pathways that may act as modulators in the TNF signaling pathway (Figure 6).

Finally, the clinical importance of exosome research in tumorigenesis, inflammatory, and chronic diseases lies in the growing understanding that molecular alterations rather than a single exosome perse identified through whole-transcriptome meta-analyses of circulating components from the oral–gut–lung axis are central to disease progression and patient outcomes. Advances in omics sciences have provided powerful tools to identify these molecular changes comprehensively and systematically. The integration of multi-omics approaches allows researchers to discover non-invasive biomarkers that can be applied for early diagnosis, prognosis, and therapeutic monitoring [189]. This shift toward minimally invasive molecular profiling enables personalized medicine strategies, improving disease management and overall survival in patients with cancer and other chronic disorders [24]. In future studies, we aim to integrate multi-omics data with clinical variables to uncover composite signatures capable of predicting outcomes in biologically heterogeneous patients and refining risk stratification.

## 4. Materials and Methods

An advanced search was performed for transcriptomic analysis on the NCBI GEO database to identify studies that analyzed coding mRNA and non-coding RNA levels with expression profiling by array or by high throughput sequencing in exosomes of healthy controls and cases related to pulmonary arterial hypertension (PAH), crohn’s disease (CD), ulcerative colitis (UC), lung cancer (LC), gastric cancer (GC) and colon cancer (CC) of related tissues and whole peripheral blood, plasma or serum. There are already several exosome studies, mostly in cell lines and patients before and after certain treatment, only a few with the parameters established, but none of them for inflammatory diseases. Three datasets were analyzed, one for every type of cancer. GSE191209 examined the total RNA of blood serum exosomes taken from non-small cell lung adenocarcinoma patients and healthy controls [190], GSE165394 examined the total RNA of whole blood exosomes taken from GC patients and healthy controls [191], and GSE71008 examined the total RNA of blood plasma exosomes taken from CC patients and healthy controls [192]. Raw count matrices were downloaded from each dataset. For each study, differential expression analysis was performed using the DESeq2 package (v1.46.0) in R (v4.5.1), estimating variance-mean dependence with a negative binomial distribution model. Genes with low counts (<10 in at least 50% of samples) were filtered out. Log_2_ fold changes (log_2_FC) and adjusted *p*-values (padj) were obtained for overexpressed RNAs (log_2_FC > 0.584, padj < 0.05). Gene identifiers were harmonized (e.g., Ensembl to HGNC) using the biomaRt package (v2.64.0) to ensure compatibility across datasets. For the meta-analysis statistical approach, the results were integrated using the MetaVolcanoR package (v1.4.0) in R, which combines log_2_FC and *p*-values from independent studies. A random-effects model was applied to identify consistently overexpressed RNAs across the three cancer types, accounting for heterogeneity (e.g., differences in cancer types and sample sources). A combined meta-log_2_FC and meta-*p*-value was calculated using “vote-counting” and “combining *p*-values” methods (e.g., Fisher’s method). Heterogeneity was assessed with the I^2^ statistic (low: <25%; moderate: 25–50%; high: >50%) and Tau^2^. The functional annotation analysis of upregulated RNAs of the metanalysis was performed by LNCSEA 2.0 (http://bio.liclab.net/LncSEA/index.php (acceded on 14 June 2025)) [193], aiming to provide a more comprehensive set of functional lncRNAs and enhanced enrichment analysis capabilities, covering numerous regulatory data sets, including lncRNA-related transcription co-factor binding, chromatin regulator binding, and chromatin interaction data, and DAVID mostly for coding mRNAs [194].

GENIE3 (GEne Network Inference with Ensemble of trees) is an algorithm based on variable selection with ensembles of regression trees, used to construct gene regulatory networks (GRNs) of inflammatory and tumoral processes to predict the expression pattern of a target genes of every key TF identified in the transcriptomic analysis of LC, GC and CC related exosomes in peripheral blood and related tissues, according to the expression patterns of all the other genes (common overregulates genes in each inflammatory and tumoral disease), using Random Forests or Extra-Trees-based ensemble methods [195]. The GRNs were constructed with the common overregulated genes expression matrix and TFs of the TRNs, using GSE113439 for PAH, GSE75214 for CD, GSE92415 for UC, GSE19804 for LC, GSE9348 for GC, and GSE63089 for CC, and R library BioNERO: an all-in-one R/Bioconductor package for comprehensive and easy biological network reconstruction [196]. ncFANs v2.0 was used to construct and predict the LncRNA-centric regulatory network of blood exosomes protein-coding and non-coding RNAs TRNs (cncTRN) related to the control of gut and lung tumorigenic processes, based on a total of 11 356 ChIP-seq datasets involved in 1354 TFs obtained from the Cistrome database, establishing the relationship between regulatory non-coding RNAs along with protein-coding genes (PCG) as transcription factors (TFs), and visualized with Cytoscape Version 3.10.3 [197]. The ChIP-X Enrichment Analysis 3 (ChEA3) was used to predict cncTRNs of regulatory RNAs guiding the formation of inflammatory and tumoral TRNs [198].

The results section focuses on the gene annotation analysis of the specific overregulated RNAs related to extracellular exosomes from blood plasma, and serum related to LC, GC and CC patients, and those identified for every inflammatory and tumoral disease of gut and lung in our previous transcriptional analysis, highlighting the key biological function of protein-coding and non-coding RNAs in the regulation of gene expression and signaling pathways related to microbiota and membrane receptors crosstalk, the regulation of genomic and epigenomic mechanisms of gene expression, and biological function involved the unlocking of phenotypic plasticity during the acquisition of the hallmarks of cancer in the oral–gut–lung axis. We focus on protein-coding and non-coding transcriptional regulators related to gut and lung extracellular exosomes function in tumoral and inflammatory diseases, to analyze the control of TFs and LncRNAs carried out by cancer exosomes through blood circulation in the construction and regulation of TFs belonging to TRNs of gut and lung cells passing by inflammatory and tumoral processes. To contextualize and experimentally validate the biological function of the differentially expressed protein-coding and non-coding RNAs identified in the meta-analysis and the analysis of TRNs, the artificial intelligence language model Gemini (Google) was used. Gemini was used to perform advanced and systematic searches of scientific literature, focusing on experimental studies (in vitro and in vivo) that validated the function of key genes (TFs and lncRNAs) in exosomal communication, microbiota-host cell crosstalk, and their role in inflammation and tumorigenesis along the oral–gut–lung axis. This tool facilitated the compilation of experimental evidence supporting the role of these RNAs in gene regulation, signaling pathways, and phenotypic plasticity, critical elements for the construction of the discussion section. Thus, a transcriptomic analysis was made based bioinformatic analyses of high sequencing gene expression studies to be able to organize and present all the scientific evidence present in several publications in the field of microbiome, inflammatory and tumoral diseases of the oral–gut–lung axis, and the actual evidence of the biological processes and signaling pathways that might be related to extracellular exosomes cargo function. In the conclusion section, we summarized the key RNAs involved in biological processes and signaling pathways related to host–microbiota communication and transcriptional regulation in every disease, to establish the current state of the art in exosomes and the establishment of inflammatory and tumorigenic diseases research, as the guiding core and starting point for future multiomics studies in this field.

## 5. Conclusions

The complex regulatory interactions between human cells and microbiota involve exosomes that function as transporters of mRNA-coding and non-coding RNAs. Exosomes transport, through the blood stream, RNAs that codify membrane receptors related to the interaction of microbiota with LC, GC, CC, PAH, CD, and UC cells, mainly SLC proteins, integrins, syndecans, and glycoproteins, as core communication signals, which are key deregulated genes in cancer and inflammatory diseases of the gut and the lung transported by exosomes. The literature experimentally validates that transporters of the SLC family (SLC2A1, SLC3A2) interact physically and functionally with viruses such as HTLV and HCV, as well as with bacteria and parasites (Ehrlichia, Plasmodium), facilitating cell entry and metabolic reprogramming of the host. Simultaneously, integrins (ITGB4, ITGA5) and syndecans (SDC1) act as critical adhesion and invasion receptors for various pathogens, including Zika, HPV, Streptococcus, and Listeria, promoting the creation of infectious niches and favoring metastasis. In the immunological context, receptors such as CD44, CD47, and ICAM-1 are co-opted by agents such as *H. pylori*, *P. gingivalis*, and *P. falciparum* to mediate tissue adhesion, evasion of phagocytosis, and vascular obstruction. Finally, signaling molecules such as WNT5A, MET, and NETO2 are modulated by bacterial and viral infections (including SARS-CoV-2 and *M. tuberculosis*) to alter cell survival, inflammation, and proliferation pathways, reinforcing the pathogenic role of these exosome-borne receptors. Some have no direct experimental evidence of their interaction with microorganisms; however, most of them are related to several cellular processes and signaling pathways in inflammation and cancer. Exosomes also transport through the blood stream RNAs that codify transcriptional regulators that belong to inflammatory and tumoral TRNs, as regulatory and communication signals transferred via exosomes between the oral cavity, gut, and lungs, potentially explaining microbial community influences inflammation and cancer as a gene network across these sites. The transcriptomic analysis of lung and gut exosomes helped to identify key regulatory molecules within exosomes that drive tumorigenesis or inflammation along oral–gut–lung axis, revealing key non-invasive biomarkers for early detection and possible therapeutic targets. The construction and analysis of these cncTRNs in exosomes from the oral–gut–lung axis represents a new global approach to unraveling the complex interplay between inflammation, microbiota, and cancer. The whole transcriptome meta-analysis of blood exosomes for lung and gut cancer identified shared and unique molecular signatures among these three tumor types, defining a robust and consistent patterns of mRNA-coding and non-coding RNAs, that may interact forming complexes related to gene expression regulation, that may serve as noninvasive biomarkers for cancer detection, diagnosis, or prognosis, that are similarly regulated in the three types of cancer. Transcriptomics technologies allowed us to identify mRNAs, lncRNA and pseudogenes which interact to accomplish regulatory functions involved in the acquisition of the hallmarks of cancer, and travel along the oral–gut–lung axis through circulating exosomes. The direct link to host–microbiota interaction via exosomal mRNA-coding and non-coding RNAs must be more extensively studied for every population in the context of complex inflammatory and tumoral diseases, to identify and specifically target the fundamental mechanism of microbiota and host-derived exosomal RNAs in maintaining homeostasis and responding to dysbiosis.

## Figures and Tables

**Table 1:** Cellular processes regulated by gene targets of TFs transported by cancer-related exosomes in peripheral blood, part of tumoral TRNs in the related tissue.

Tissue	TF	Blood	Gene Targets Cellular Processes
LC	FOXE1	GC	Biological processes: chromosome segregation, DNA repair, CMG and MCM complex, DNA damage, extracellular exosome, cell proliferation, RNA binding, apoptosis, homologous recombination, cell junction, and RNA splicing. Signaling pathways: DNA replication; base excision repair; mismatch repair; p53, pyrimidine, purine, and glutathione metabolism; nucleotide excision repair; nucleotide metabolism; cellular senescence; small cell LC; HTLV-1, EBV, and HPV infection; viral carcinogenesis; microRNAs; and pathways in cancer. Epigenetic reprogramming: Phosphoprotein, acetylation, Ubl conjugation, histone kinase activity, histone binding, protein modification process, methylation, prenylation, and hydroxylation.
	HDGF	LC	
GC	BHLHE40	LC	Biological process: Collagen-containing extracellular matrix, integrin binding, cell surface, cell adhesion, differentiation, migration, division, proliferation, migration, junction, and projection, extracellular exosome, angiogenesis, apoptosis, receptor ligand activity, DNA replication, membrane raft, and host cell receptor for virus entry Signaling pathways: ECM–receptor interaction, focal adhesion, protein digestion and absorption, cell cycle, amoebiasis, PI3K-Akt, HPV, cytokine-cytokine receptor interaction, malaria, phagosome, small cell LC, IL-17, response to type II interferon, NF-kappa B, and KSHV infection. Epigenetic reprogramming: Glycoprotein, hydroxylation, phosphorylation, protein homodimerization activity, histone acetyltransferase binding, and protein ubiquitination.
	CEBPB	LC	
	YBX3 TEAD4	LC	
CC	CEBPB	LC	Biological process: Protein binding, cell division, collagen-containing extracellular matrix, RNA binding and splicing, nucleotide-binding, ATP binding, mRNA 5′-UTR binding, transcription coregulator binding, DNA replication, rRNA processing, cell surface, extracellular exosome, host–virus interaction, translation, apoptosis, cell migration and proliferation, EMT, DNA damage response, ribosome biogenesis, and stem cell factor receptor activity. Signaling pathways: Cell cycle, ECM–receptor interaction, nucleocytoplasmic transport, HPV infection, cellular senescence, focal adhesion, small cell LC, EBV infection, proteoglycans in cancer, pathways in cancer, malaria, hippo HPC, amoebiasis, influenza A, insulin receptor, lysosome, chemokine-mediated, WNT, and VEGF receptor-1. Epigenetic reprogramming: Acetylation, Ubl conjugation, phosphoprotein, histone binding, protein digestion and absorption, methylation, histone kinase activity, and histone phosphatase activity.
	GTF3A	LC	
	JADE3	CC	
	JUN	LC	
	MAZ	LC	
	MYC	LC	
	NFE2L3	GC	
	SOX9	LC	
	TGIF2	LC	
	ZEB1	LC	
	ZNF503	GC	

**Table 2:** Cellular processes regulated by gene targets of TFs transported by cancer-related exosomes in peripheral blood, part of inflammatory TRNs in the related tissue.

Tissue	TF	Blood	Gene Targets Cellular Processes
PAH	IRF9	LC	Biological process: Extracellular exosome, transmembrane helix, cell junction, adhesion, migration and growth, cytoplasmic vesicle, Golgi apparatus, membrane raft, angiogenesis, nuclear inner membrane, mitochondrial ribosome binding, retromer complex binding, and innate immunity. Signaling pathways: HPV, hepatitis C, salmonella, vibrio cholerae and epithelial cell signaling in Helicobacter pylori infection, leukocyte transendothelial migration, endocrine resistance, efferocytosis, mTOR, phagosome, VEGF, NOTCH, autophagy, neurotrophin, sphingolipid, thyroid hormone, central carbon metabolism in cancer, inositol phosphate metabolism, lysosome, chemokine, FoxO, aldosterone-regulated sodium reabsorption, phosphatidylinositol signaling system, PD-L1 expression and PD-1 checkpoint pathway in cancer, EGFR tyrosine kinase inhibitor resistance, chemical carcinogenesis—reactive oxygen species, pathways and microRNAs in cancer, and cell surface receptor. Epigenetic reprogramming: Glycoprotein, phosphoprotein, prenylation, negative regulation of protein phosphorylation, protein dephosphorylation, and histone phosphatase activity.
	GLMP	GC	
	MAF	LC	
	ZNF346	GC	
	ZNF503	GC	
CD	FOXJ3	LC	Biological processes: Transit peptide, mitochondrion, transmembrane helix, protein transport, and extracellular exosome and endosome. Signaling pathways: Multiple metabolic pathways, fatty acid degradation, citrate cycle (TCA cycle), thermogenesis, peroxisome, Rap1, drug metabolism—cytochrome P450, phosphatidylinositol signaling system, PPAR, biosynthesis of cofactors, valine, leucine and isoleucine degradation, chemical carcinogenesis—receptor activation, inflammatory mediator regulation of TRP channels, proteoglycans in cancer, ErbB, phospholipase D, longevity regulating, calcium, PI3K-Akt, endocrine resistance, MAPK, ABC transporters, tight junction, oxidative phosphorylation, gastric acid secretion, and virion—hepatitis viruses. Epigenetic reprogramming: Acetylation, phosphoprotein, protein glycosylation, protein dephosphorylation, and histone phosphatase activity.
	OVOL2	GC	
	ZNF395	LC	
UC	ELF4	LC GC	Biological process: Mitochondrion, transit peptide, extracellular exosome, nucleotide-binding, ATP-binding, cellular respiration, projection, differentiation and detoxification, endosome, transmembrane transporter activity, transmembrane helix, membrane raft, protein transport, and phosphatidic acid binding. Signaling pathways: Multiple metabolic pathways, valine, leucine, and isoleucine degradation; citrate cycle (TCA cycle); PPAR; peroxisomes; thermogenesis; biosynthesis of cofactors; phosphatidylinositol signaling system; calcium; cAMP; AMPK; aldosterone synthesis and secretion; Ras; Rap1; oxidative phosphorylation; and ErbB. Epigenetic reprogramming: Acetylation, phosphoprotein, protein phosphorylation, histone kinase activity, and protein dephosphorylation.
	FOXJ3	LC	
	HNF4G	LC	
	HOXA5	CC	
	MXI1	LC	
	NR1H4	LC	
	NR1I2	GC	
	OVOL2	GC	
	ZNF350	GC	
	ZNF395	LC	

## Data Availability

The transcriptomics datasets analyzed in this study can be found in the GEODatasets repository of the NIH. The original contributions presented in the study are included in the article/Supplementary Material; further inquiries can be directed to the corresponding author.

## Supplementary Materials

The following supporting information can be downloaded at https://www.mdpi.com/article/10.3390/epigenomes9040052/s1: Table S1: Lung cancer exosomes (LCEXO) annotation and network analysis. Table S2: Gastric cancer exosomes (GCEXO) annotation and network analysis. Table S3: Colon cancer exosomes (CCEXO) annotation and network analysis. Table S4: The whole transcriptome meta-analysis of lung, gastric, and colon cancer circulating exosomes. Table S5: Common RNAs analysis between blood and tissue of patients with pulmonary arterial hypertension (PAH), Crohn’s disease (CD), ulcerative colitis (UC), lung cancer (LC), gastric cancer (GC), and colon cancer (CC). Table S6: Inflammatory diseases gene regulatory network (GRN) analysis.
